# Biparametric score as a new tool for early indication of surfactant in preterm infants

**DOI:** 10.1016/j.jped.2025.101425

**Published:** 2025-08-18

**Authors:** Ana Román Fernández, Jessica Gómez Ávila

**Affiliations:** Hospital Universitario Virgen Macarena, Neonatology Department, Sevilla, Spain

**Keywords:** Lung ultrasound, Preterm infants, Respiratory distress, Surfactant treatment

## Abstract

**Objective:**

To investigate whether the use of a biparametric score, based on lung ultrasound (LUS) and oxygen saturation/fraction of inspired oxygen ratio (SF ratio), in preterm infants with respiratory distress syndrome (RDS) allows earlier surfactant therapy (first 3 hours of life) compared to classic FiO_2_ criteria.

**Material and methods:**

Before-after design study, performed in a tertiary neonatal intensive care unit. Inclusion criteria were newborns with gestational age < 34 weeks with clinical RDS and respiratory support with noninvasive ventilation. The patients were divided into two groups, the control group, with surfactant indication according to classic criteria, collected retrospectively, and the new protocol group, with surfactant criteria according to biparametric score.

**Results:**

61 patients were included. The new protocol group received surfactant earlier (all patients in the first 3 hours, p 0.013). Likewise, after surfactant treatment, newborns in this group required lower FiO_2_ (p 0.001) and a better pulmonary ultrasound evolution according to LUS (p 0.008).

**Conclusions:**

Biparametric scoring allowed earlier surfactant therapy and reduced post-treatment oxygen requirement. This protocol offers a more personalized approach tailored to the patient's needs, which helps us in decision-making.

## Introduction

Primary surfactant deficiency or respiratory distress syndrome (RDS) continues to be an important cause of morbidity and mortality in very preterm infants. Continuous positive airway pressure and surfactant are the first- and second-line treatments, respectively, in this pathology.

There is evidence that the administration of surfactant in the first hours of life decreases mortality and the risk of developing BPD compared to when it is administered later, [1] so early identification of newborns who may benefit from this treatment, which is not free of complications, is a challenge in neonatal critical care. However, the optimal criteria for its administration are not entirely clear.

The different international guidelines recommend the administration of surfactant in preterm newborns, establishing a FiO_2_ threshold. In Europe, the most widespread indication is to administer it as soon as possible when FIO_2_ > 30 % is required to maintain oxygen saturations adequate for gestational age after optimizing noninvasive ventilation, preferably by noninvasive techniques [2]. These recommendations have weak evidence and are based on retrospective studies that evaluate the predictors of noninvasive ventilation failure, one of which is FIO_2_ [3,4].

However, studies that support the use of lung ultrasound as a predictive tool for the need for early surfactant replacement have much more robust evidence, with a larger number of patients, including some meta-analyses [5,6]. They are based on the use of a semiquantitative score that quantifies the loss of lung aeration (LUS: Lung Ultrasound Score) [7, 8, 9, 10, 11]. In fact, the latest European Consensus Guideline for the management of RDS 2 includes “compatible lung ultrasound” without further specification as a possible criterion for indicating treatment with surfactant.

As demonstrated by Brusa et al., [12] there is good interobserver agreement when interpreting neonatal lung sonography images, with even higher concordance observed in newborns with respiratory distress syndrome compared to other pulmonary conditions.

On the other hand, the ability of the oxygen saturation/fraction of inspired oxygen ratio (SF ratio) as a parameter to guide surfactant treatment in preterm newborns has been analyzed in isolation or in combination. SF ratio is a non-invasive marker that gives a good correlation with PF ratio (arterial O_2_ pressure/FiO_2_) when oxygen saturation is between 92 and 98 %. Recent publications show that LUS and SF ratios are good predictors for surfactant treatment [13]. The combination of these two parameters shows a higher predictive value for the need for surfactant, as supported by a multicenter observational cohort study conducted in Italian neonatal units in infants younger than 34 weeks, regardless of the degree of prematurity [14].

## Material and methods

A before-after design study was carried out in a tertiary-level hospital integrated into the Spanish health system with a level IIIB Neonatal Unit attending around 2500 deliveries per year.

All the professionals working in the Unit had received certified training in lung ultrasound prior to the study. A Gehealthcare LOGIC S7 Xdclear 2.0 ultrasound machine with an L8–18i linear probe suitable for this type of patient was used.

The study was approved by the hospital Ethics Committee, and written informed consent was requested from the legal guardians of the participants prior to inclusion.

### Objectives

The main objective of the study was to determine if a diagnosis of RDS guided by a biparametric test allowed for early surfactant in preterm infants younger than 34 weeks, compared to the classic criteria for surfactant treatment.

The secondary objectives were to determine the number of patients who receive surfactant, how many receive it in the first 3 hours of life, the subsequent respiratory evolution and the need for mechanical ventilation in the following 72 hours after surfactant treatment in both groups, and to determine the SF ratio 30 min after surfactant administration in the new protocol group.

### Intervention

A biparametric scoring protocol was developed as the main intervention, integrating Lung Ultrasound Score (LUS) and SF ratio to guide surfactant administration decisions.

### Methodology

The patients were divided into two groups.

• A control group, consisting of preterm newborns under 34 weeks with a diagnosis of RDS and need for noninvasive ventilation, in whom the administration of surfactant was assessed according to the classic criteria of the European Consensus Guideline 2 for the treatment of neonatal RDS in preterm infants, collected between January 2021 and June 2022.
• A new protocol group, recruited between July 2022 and March 2024, consisting of preterm infants younger than 34 weeks with clinical signs of RDS and the need for noninvasive ventilation in whom the need for surfactant administration was assessed according to the new protocol implemented in the unit based on lung ultrasound assessment and SF ratio.

All preterm newborns included in the study were initially stabilized using non-invasive ventilation (NIV), with support pressures ranging between 5 and 7 cmH₂O. This respiratory support was applied upon admission to the unit, maintaining standardized parameters to ensure adequate oxygenation and to avoid early intubation, in accordance with current neonatal respiratory management protocols.

For the implementation of this protocol, a preliminary statistical analysis of the results of the initial cohort (control group) was carried out, where the authors performed lung ultrasound prior to the administration of surfactant, without this being a determining factor when indicating this treatment, and the results obtained in other similar studies were taken into account. It was concluded that having more than 7 points in this score in the first lung ultrasound increased the need for surfactant by 81 % with respect to those with less than 7 points, with a Hazard ratio of 0.19 (0.04–0.75) p 0.02.

The ultrasound study was carried out following the Brat [15] Ultrasound Score, exploring 3 zones in each hemithorax (upper anterior, lower anterior and lateral) and giving a score from 0 to 3 points in each lung field, obtaining a final score between 0 and 18 points.

**Fig. 1:**
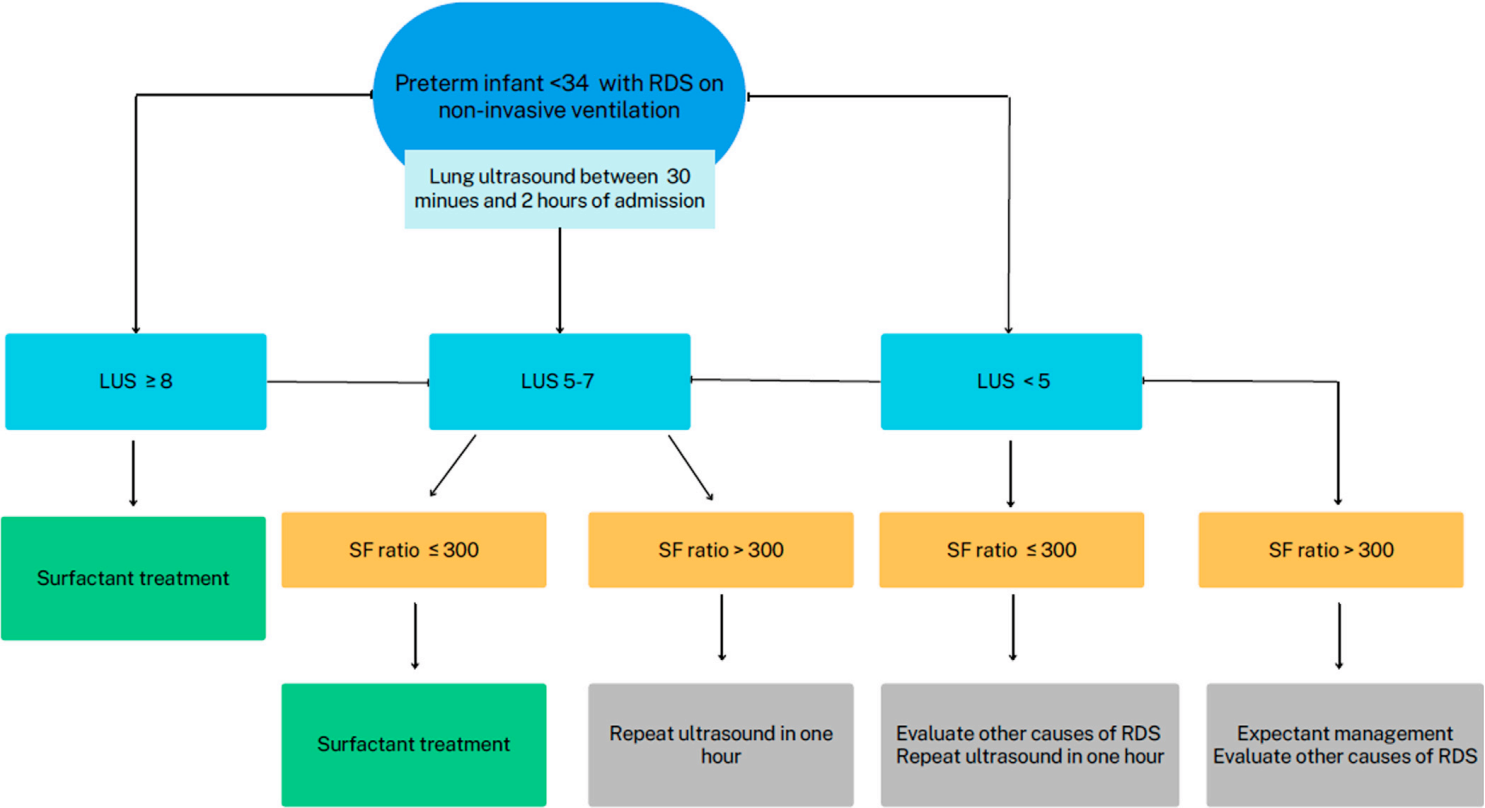
Algorithm for decision-making regarding surfactant administration.

The new protocol for the administration of surfactant in the unit was based on the performance of lung ultrasound in preterm infants under 34 weeks with clinical signs of RDS and noninvasive ventilation between the first thirty minutes and two hours of admission, proceeding as shown in the algorithm in Fig. 1.

The exclusion criteria in both groups were newborns with major malformations; with severe sepsis or septic shock; with suspected pneumothorax or meconium aspiration syndrome; exitus in the first 72 hours of life; newborns having been administered surfactant prior to lung ultrasound; for refusal by the legal representatives to participate in the study are excluded.

The administration of surfactant (Curosurf 200 mg/kg/dose) is preferably performed by a minimally invasive technique and using comfort measures in all patients at the time of application.

### Statistical analysis

A statistical study was carried out where the distributions were analyzed, according to the normality or non-normality of the sample, using the Kolmogorov-Smirnov or Shapiro-Wilk tests. Qualitative variables were expressed as frequency (percentage, %), and continuous quantitative variables were expressed as median with interquartile range (IQR) [p25-p75] or as mean ± standard deviation (SD). The relationship between qualitative variables was calculated using Pearson's Chi-square test, and between quantitative variables, the Student's *t*-test and Mann-Whitney *U* test according to the distribution of the data. The McNemar test or the Wilcoxon test was used to test the hypothesis of paired data. A significance level below 5 % was established.

## Results

A total of 61 patients were included, 30 in the new protocol group and 31 in the control group. No patient was excluded because of exitus in the first 72 hours of life or because family members did not agree to participate in the study. An analysis of the characteristics of both populations was carried out, finding similar characteristics, except for a difference in the mean gestational age of 9 days between the two groups, which could be up to 17 days longer in the control group. When stratifying by gestational age, a greater number of patients in the 31–33+6 weeks group were observed in the control group (Table 1).

**Table 1:** Demographic variables of the population. Data expressed as mean (standard deviation), median (p25-p75), or number ( %) as appropriate.

	New protocol group (*n* = 30)	Control group (*n* = 31)	p
**Female**	12 (40 %)	9 (29 %)	NS
**Birth weight** **(grams)**	1240 (953–1540)	1330 (1060- 1840)	NS
**Caesarean delivery**	19 (63,3 %)	21(67,7 %)	NS
**Gestational age (weeks)**	30+1 (27+4 - 31+2)	31+3 (28+5 - 33)	p 0018
**< 28**	8 (26,7 %)	4 (12,9 %)	NS
**28–30+6**	13 (43,3 %)	7 (22,6 %)	NS
**31–33+6**	9 (30 %)	20 (64,5 %)	0026
**Antenatal steroids (≥ 2 doses)**	22 (73,3 %)	24 (77,4 %)	NS

* NS, not significant.

Lung ultrasound was performed in all patients included in the study, with a statistically significant difference (p 0.016) in the timing of this assessment, with greater dispersion in the control group (Table 2). The mean score of the first ultrasound, according to the Brat ultrasound score, in the patients included in the control group was somewhat lower than in the new protocol group (5.77 points SD 4.6 vs. 7.67 points SD 4.2).

**Table 2:** Analysis of total patients. Data expressed as mean (standard deviation), median (p25-p75) or number ( %) as appropriate.

	New protocol group (*n* = 30)	Control group (*n* = 31)	p
**Maximum FiO_2_ before assessing surfactant treatment**	30 (21–35)	25 (21–45)	NS
**Initial pCO_2_**	54 (45,7–58,2)	50,8 (42,3–57)	NS
**Hours of life 1st ultrasound**	2 (1,3–2)	2 (1–3)	p 0016
**1st ultrasound score**	7,67 (4,2)	5,77 (4,6)	NS
**Surfactant administration**	18 (60 %)	15 (48,4 %)	NS
**Days on noninvasive ventilation**	5 (2–11)	1 (1–3)	NS
**Bronchopulmonary dysplasia**	8 (28,6 %)	5 (17,2 %)	NS

*NS, not significant.

In the control group, surfactant was administered in 48.4 % of the patients, and in the new protocol group, in 60 % of them, without statistically implying that the new way of deciding the administration of surfactant increased the number of patients treated (Table 2).

In the new protocol group, all the patients who received surfactant had an initial ultrasound score of more than 7 points. The mean Brat ultrasound score in patients who received surfactant prior to surfactant administration in the new protocol group was 10.78 points (SD 1.5) and in the control group, 8.33 points (SD 5.1). When pre-treatment FiO_2_ was analyzed in these patients, 7 (38.8 %) in the intervention group required a FIO_2_ ≤ 30 % vs 3 (20 %) in the control group.

In the new protocol group, 100 % of the patients received surfactant in the first 3 hours of life, compared to 66.7 % in the control group, with a statistically significant difference (p 0.013).

When FiO_2_ was assessed one hour after administering the treatment in both groups, 88.9 % of the patients in the new protocol group required FiO_2_ below 30 % compared to 73.3 % in the control group. On analyzing the FiO_2_ trend in patients receiving treatment according to the new protocol, the number of patients requiring a FiO_2_ was found to be greater than or equal to 30 % dropped from 72 % to 11 % after treatment. In the control group, this difference is smaller, going from 80 % to 26.7 %, obtaining statistically significant differences (p 0.001). A statistically significant difference in the ultrasound evolution was also observed when analyzing the change in the pre-post surfactant administration score in favor of the new protocol group (p 0.008). In this group, the LUS median was initially 11 points, lowering 48 hours later to 2 points. The improvement was less in the control group, changing from a median of 8 to 3 points. In the rest of the clinical evolution parameters, there were no differences between the two groups (Table 3).

**Table 3:** Analysis of patients receiving surfactant. Data expressed as mean (standard deviation), median (p25-p75) or number ( %) as appropriate.

	New protocol group (*n* = 18)	Control group (*n* = 15)	P
**Maximum FiO_2_ before assessing surfactant treatment**	30 (25–40)	40 (30–50)	NS
**Surfactant administered in the first 3 hours of life**	18 (100 %)	10 (66,7 %)	p 0.013
**FiO_2_ < 30% after 1 hour surfactant**	16 (88,9 %)	11 (73,3 %)	NS
**Failure of non-invasive ventilation after 72 hours**	2 (11,1 %)	2 (13,3 %)	NS
**Days on noninvasive ventilation**	5 (3–7)	3 (2–5)	NS
**Total respiratory support days**	18 (6–32)	6(5–43)	NS

*NS, not significant.

In the patients in the new protocol group, the SF ratio was evaluated before and after surfactant treatment, and an average difference of 100 units (CI 58–140) was observed in the subsequent SF ratio (*p* < 0.001).

After applying the new protocol, only 3 patients (10 %) obtained an ultrasound score between 5–7 points, requiring the SF ratio to assess the attitude to follow. All of them presented an SF ratio> 300, and surfactant administration was not indicated. None of them presented worsening in ultrasound aeration, nor did they subsequently receive surfactant. Thirty percent of the patients in the new protocol had scores below 5 points and an SF ratio >300.

## Discussion

The present study reinforces the evidence previously provided by the literature [5,6,8,10,15] that lung ultrasound is a useful tool for guiding surfactant administration.

Although lung ultrasound has become a standard technique in the diagnosis of RDS in the newborn, there is variability among the lung zones assessed, the way of assigning the LUS score, and the cut-off points used to indicate surfactant treatment in the different studies mentioned. However, the differences found are minimal, and the clinical implications are negligible, as demonstrated by a recent multicenter study comparing the three most commonly used ultrasound scores [16]. All scores had an excellent ability to predict the need for surfactant and optimal intra- and interobserver agreement. In the unit, the authors use the Brat score, assuming that these patients have a homogeneous deficit and that exploring posterior fields does not provide significant extra information that would justify delaying or complicating the technique.

In relation to the timing of the first ultrasound, the authors emphasize the importance of allowing sufficient time for the physiological transition mechanisms to take place and to ensure good recruitment with noninvasive ventilation. Therefore, in order to be able to detect when noninvasive ventilation is insufficient, the authors do not recommend performing the first ultrasound evaluation of pulmonary aeration in the delivery room or immediately after transfer to the neonatal unit, unless the objective is other than deciding whether or not to administer exogenous surfactant.

As in the randomized clinical trial by Rodriguez-Fanjul et al., [11] the use of the new protocol has been shown to achieve treatment earlier in the course of the pathology in question, reducing the time to administer the first dose of surfactant. All the patients included in the intervention group received treatment within the first three hours of life, while in the control group, 33 % of the patients were treated later. Although the authors have not been able to demonstrate this in the present study, it is possible that this results in less need for subsequent mechanical ventilation and better respiratory outcomes as reported in the literature.

Although the authors know that ultrasound scores that assess pulmonary aeration have been shown to correlate well with the degree of oxygenation, the inclusion of the SF ratio is of interest in patients in the gray zone. The authors refer to the gray zone as that which includes scores between 5 and 7 points, in which lung ultrasound alone may be insufficient because it corresponds to intermediate lung patterns. Thus, the proposed biparametric score has the potential to assist in therapeutic decision making in patients in whom the decision to administer surfactant may be more controversial. Studies claim that the classic criteria for surfactant administration, based primarily on FiO_2_, may be arbitrary and may not accurately reflect patient oxygenation [11,14]. On the other hand, De Luca suggests that the use of the SF ratio to predict the need for surfactant could be circular reasoning, since FiO_2_, being a component of this index, is also a determining factor in the final decision [17]. For this reason, the combination of lung ultrasound together with the SF ratio could be the best predictor to assess administration by providing an earlier and more accurate assessment, allowing a timelier intervention and potentially better outcomes, as this multimodal approach provides information on lung structure and on oxygenation efficiency. This was demonstrated by Raimondi et al. in demonstrating an area under the curve (AUC) combining lung ultrasound and SAFI of 0.93, significantly exceeding that of lung ultrasound or SAFI alone [14].

One of the most widespread concerns is whether the use of ultrasound alone to indicate surfactant treatment could lead to an increase in the number of patients treated. In this case, the use of the biparametric score did not significantly increase this n, and the same is the case in most studies published in recent years, which show how lung ultrasound can detect surfactant deficiency earlier without necessarily increasing the total number of patients treated [8,10,11]. Of the 18 patients who received surfactant in the new protocol group, the authors found that up to 38 % of them would not have been treated if their evaluation had been based exclusively on FiO_2_, risking a worse evolution, longer exposure time to oxygen, and its toxic effects.

In the present study, when analyzing the evolution of the patients who received surfactant in both groups, the authors found a more significant overall improvement in the new protocol group due to greater progress in ultrasound scores 48 hours later, even though the initial scores were worse, and a statistically significant superior decrease in FiO_2_ after one hour of surfactant administration. Therefore, the present study supports the superiority of combined assessment for treatment decision [14,18].

The use of the protocol did not lead to an increase in the need for invasive mechanical ventilation 72 hours later, nor was it related to an increase in the need for administration of a second dose of surfactant in these patients. However, like other authors, the authors were unable to demonstrate a direct reduction in the number of total days of ventilation or in the development of bronchopulmonary dysplasia (BPD).

The before-and-after design used to evaluate the effect of introducing the new protocol may represent a limitation, as it does not account for possible secular trends. However, the authors consider that the bias is minimal given the short evaluation period and the absence of other changes in clinical protocols and local epidemiology. One of the main limitations of this study lies in the impossibility of performing a prior sample size calculation. The comparison was made between a historical cohort and a prospective cohort, determined by the number of admissions during the established periods. Since this was an implementation of a unit-wide protocol, all patients who met the inclusion criteria were consecutively included, without randomization or predetermined sample size calculation.

This biparametric score is not applicable to intubated patients, since, as indicated in the study by Bouhemad et al., [19] mechanical ventilation may modify the pulmonary echographic pattern, attributed to the higher mean airway pressure, and therefore may not be a good predictor for the need for surfactant as the authors find falsely lower scores.

In conclusion, the present study develops a feasible predictive model for early surfactant therapy in newborns < 34 weeks with RDS, offering a more personalized approach tailored to the patient's needs, which helps us in clinical decision making. It allowed earlier surfactant therapy, it reduced post-treatment oxygen requirement, and it achieved improved lung aeration sonographically compared to classical criteria.

The authors believe that conducting multicenter prospective cohort studies would allow for the validation of the score in a larger population and the evaluation of other long-term clinical outcomes, such as the development of bronchopulmonary dysplasia. From the authors’ point of view, a randomized trial could be considered unethical at present, since lung ultrasound is already the imaging test of first choice for respiratory pathology in NICUs.

## Sources of funding

This study has not received any funding whatsoever. The authors have received a research award from the Spanish Society of Neonatology (Jose Quero grant 2023).

## Ethical approval

All procedures performed in this study involving human participants were in accordance with the ethical standards of the institutional research Committee (Ethics Committee of Virgen Macarena and Virgen del Rocio University Hospitals) and the 1964 Declaration of Helsinki and its subsequent amendments or comparable ethical standards.

## Informed consent

Informed consent was obtained from the legal guardians of all individual participants included in the study.

## Conflicts of interest

The authors declare no conflicts of interest.
